# Stress and Resilience in Functional Somatic Syndromes – A Structural Equation Modeling Approach

**DOI:** 10.1371/journal.pone.0111214

**Published:** 2014-11-14

**Authors:** Susanne Fischer, Gunnar Lemmer, Mario Gollwitzer, Urs M. Nater

**Affiliations:** 1 Clinical Biopsychology, Department of Psychology, University of Marburg, Marburg, Hesse, Germany; 2 Methodology and Social Psychology, Department of Psychology, University of Marburg, Marburg, Hesse, Germany; Harvard Medical School, United States of America

## Abstract

**Background:**

Stress has been suggested to play a role in the development and perpetuation of functional somatic syndromes. The mechanisms of how this might occur are not clear.

**Purpose:**

We propose a multi-dimensional stress model which posits that childhood trauma increases adult stress reactivity (i.e., an individual's tendency to respond strongly to stressors) and reduces resilience (e.g., the belief in one's competence). This in turn facilitates the manifestation of functional somatic syndromes via chronic stress. We tested this model cross-sectionally and prospectively.

**Methods:**

Young adults participated in a web survey at two time points. Structural equation modeling was used to test our model. The final sample consisted of 3′054 participants, and 429 of these participated in the follow-up survey.

**Results:**

Our proposed model fit the data in the cross-sectional (χ^2^(21)  = 48.808, *p*<.001, CFI  = .995, TLI  = .992, RMSEA  = .021, 90% CI [.013.029]) and prospective analyses (χ^2^(21)  = 32.675, *p*<.05, CFI  = .982, TLI  = .969, RMSEA  = .036, 90% CI [.001.059]).

**Discussion:**

Our findings have several clinical implications, suggesting a role for stress management training in the prevention and treatment of functional somatic syndromes.

## Introduction

The term ‘functional somatic syndrome' (FSS) refers to various clusters of somatic symptoms (e.g., fatigue, abdominal or musculoskeletal pain) that cannot be adequately explained by means of modern medicine (‘medically unexplained symptoms'). Conditions such as chronic fatigue syndrome, fibromyalgia syndrome, or irritable bowel syndrome represent frequently occurring disorders that fall into this broad category. Functional somatic syndromes as well as medically unexplained symptoms are prevalent in the general population [1]–[3] and account for a large proportion of health care visits both in primary [4]–[6] and secondary care [7]. They cause substantial suffering in patients and lead to a considerable amount of direct (e.g., medical care) and indirect (e.g., lost productivity) costs [8]–[10].

Despite remarkable efforts in attempting to elucidate pathophysiological mechanisms in FSS, the exact determinants and processes underlying these debilitating disorders are still unknown. Current knowledge points to a number of predisposing, precipitating, and perpetuating factors, such as genetic factors [11]–[13], viral infections [14], [15], and alterations of visceral and central sensitivity [16], [17]. A prominent line of research has been dedicated to the role of stress in FSS [18], thus conceptualizing FSS as ‘stress-related disorders'. The transactional stress theory by Lazarus and Folkman [19] provides a framework encompassing several aspects of the stress concept. According to these authors, stress is understood as a person-environment interaction, involving a potentially threatening stimulus (i.e., a stressor), and a biological and psychological stress response [19]. Importantly, this interaction is mediated by complex appraisal processes within the individual, encompassing an assessment of individual resources to deal with potentially stressful events [19]. As will be outlined in the following paragraphs, there is empirical evidence for all of these theoretical aspects to play a role in FSS.

Events during childhood that are perceived as traumatic (i.e., *childhood traumas*), such as emotional, physical, or sexual abuse [20], are among the most severe stressors and have been reported in a substantial number of FSS patients [21]. Unlike mild to moderate stress, severe early life stress including childhood trauma is well-known to permanently alter the reactivity of biological stress-responsive systems in a negative manner [22]. Simliar to its biological analogue, psychological *stress reactivity*, which is defined as an individual's personal capacity or tendency to respond to stressors [23], seems to be heightened as a consequence of early life stress. For instance, abnormal birth weight reflecting an adverse prenatal environment was linked to higher levels of psychological stress reactivity as an older adult [24]. In FSS, it has been reported that patients feel more tense or stressed after a laboratory stress test, that is, they show higher levels of psychological stress reactivity [25], [26]. Decades of (mostly biological) research have documented the effects of childhood trauma on heightened stress reactivity and subsequent adverse health outcomes [22]. However, no study has ever tested whether a trauma-induced elevation of psychological stress reactivity perpetuates or even favors the development of full-blown FSS. The present study fills this gap.

Apart from the detrimental effects of childhood trauma on stress reactivity, the impact of early life stress unfolds in another fashion: in affecting *resilience*. Initial evidence for a negative association between levels of childhood trauma and resilience exists in healthy individuals [27], [28]. Resilience can be defined as a positive personality characteristic that enhances individual adaptation, including the facets of believing in one's personal competence and accepting oneself and one's life [29]. According to the transactional stress theory [19], personality characteristics, such as resilience, largely influence appraisal processes that in turn mediate the stressor-stress response relationship. From this follows that stressors and resilience are intertwined in predicting stress reactivity: an individual with high levels of acceptance of him-/herself is unlikely to lose confidence when being faced with criticsm, and vice versa. Indeed, in an experimental study using a laboratory paradigm to induce pain, healthy individuals scoring high on a resilience scale experienced less stress and even pain after being exposed to a tourniquet procedure [30]. Regarding FSS, research demonstrates comparably low overall scores in associated personality characteristics such as sense of coherence [31] and self-efficacy [32] in these patients. So far, evidence indicates that resilience mediates the effect of childhood trauma on psychological distress in apparenly healthy individuals [33] and Holocaust survivors [34]. However, we are not aware of any studies examining these relationships in FSS patients.

At this point, it remains unclear how experiences of childhood trauma, and (subsequent) alterations in stress reactivity and resilience influence the development of FSS. One possible mediating factor is the occurrence of *chronic stress*. Chronic stress is characterized by recurring episodes of stress that are often related to unsatisfied personal needs [35]. In Lazarus and Folkman's terms [19], experiencing chronic stress may be the result of a negative bias in the appraisal of stimuli, that is, to perceive ambiguous stimuli as a threat [36]. Recent data demonstrating a linkage between early abusive experiences, heightened levels of chronic stress, and premenstrual symptoms suggest a possible origin of these threat appraisals [37]. Similarly, a cross-sectional survey in a non-clinical sample found evidence for a positive relationship between stress reactivity with a measure of chronic stress [38]. Finally, another study conducted in fibromyalgia patients showed that 53% of the variance in chronic stress levels could be explained by self-esteem, self-efficacy, and social support, all of which are known to be associated with resilience [39].

Elevated levels of chronic stress have repeatedly been reported in patients suffering from FSS [40], [41]. Several mechanisms by which both traumatic and chronic stress foster the development of FSS are conceivable. At the biological level, traumatic [42] and chronic stress [43] result in the epigenetic modification of genes related to stress-responsive systems. In the long run, this causes dysregulation of the hypothalamic-pituitary axis, the autonomic nervous system, and the immune system [22], [44]. Notably, all of these physiological systems seem to be dysregulated in patients suffering from FSS [18] and might exacerbate symptoms like fatigue and pain [45]. From a psychological point of view, medically unexplained symptoms can be regarded as misperceptions and -interpretations of bodily sensations that are generated by stored memory representations [46]. According to Brown [46], these representations can have varied origin. For instance, sensorimotor and emotional concomitants of trauma exposure are an important source for their development [46]. For this reason, experiences of childhood trauma might directly be related to the occurrence of FSS (that are characterized by medically unexplained symptoms). In addition, a recent study found the interaction of stress and the perception of stress as ‘dangerous’ or ‘harmful’ to increase reportings of poor health [47]. It is thus likely that chronic stress leads to emotional arousal and accompanying bodily sensations that can be subject to misinterpretation [48].

**Figure 1:**
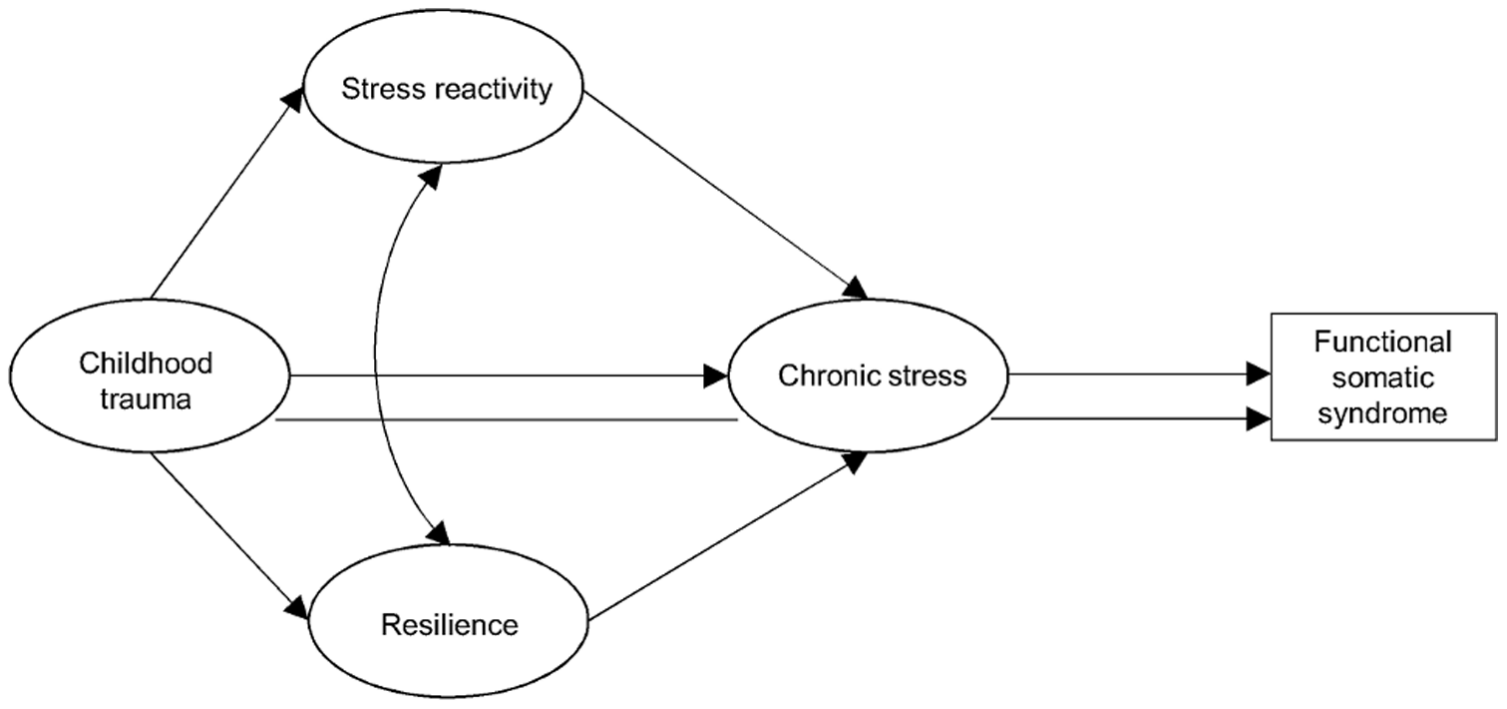
Conceptual model for FSS.

In sum, FSS seem to be associated with the occurrence of childhood trauma as well as alterations in stress reactivity at the biological and psychological level. Furthermore, chronic stress seems to play an important mediating role in translating these vulnerabilities into FSS. No study has so far targeted multiple aspects of stress (i.e., childhood trauma, stress reactivity, and chronic stress) in a large sample. Thus, the interactions between these variables remain to be specified, and we do not know of any studies that have included measures of resilience in a comprehensive model describing the role of stress in FSS. In the current study, we set out to examine the associations between childhood trauma, stress reactivity, resilience, and chronic stress in FSS in a sample of young adults. Our conceptual model depicting the hypothesized associations is illustrated in Figure 1. In brief, our model posits that the occurrence of childhood trauma takes its toll on stress reactivity and resilience, which in turn facilitate the manifestation of FSS via chronic stress. In addition, we assume that childhood trauma itself is indirectly (via chronic stress) and directly linked to FSS. Importantly, since a temporal order of events and changes in personality characteristics is implied in our model, we wanted to rule out the possibility that stress reactivity, resilience, and chronic stress were merely elevated as a consequence of having an FSS. We thus tested our model not only cross-sectionally, but also prospectively, including its evaluation in new FSS cases. We tested these hypotheses as part of a bigger study [49].

## Methods

### Sample and procedures

We have previously described how participants for this study were recruited [49]. In brief, German speaking students of Swiss colleges and universities were asked to participate in a web survey on physical and mental well-being (T_0_). In addition, the participants were asked if they wished to participate in a follow-up survey, and those who agreed were asked to complete the exact same survey six months later (T_1_). This enabled us to evaluate whether experiences of childhood trauma, heightened stress reactivity, and lower resilience were prospectively related to the occurrence of chronic stress six months later and whether this in turn predicted the development (of new) and perpetuation (of existing) FSS.

### Ethics statement

The study was conducted in accordance with the Declaration of Helsinki. The web survey design was approved by the ethics committee of the University of Zurich. Written (online) informed consent was obtained from all participants.

### Measures

We decided to shorten each scale in order to reduce the complexity of measurement models of the constructs in terms of the number of parameters to be estimated.

#### Childhood trauma

To measure childhood abuse and neglect, we used eight items from the German short version of the Childhood Trauma Questionnaire [50] that had the highest factor loadings on the general factor in this sample. The questionnaire consists of six different domains of childhood trauma: emotional abuse, physical abuse, sexual abuse, emotional neglect, physical neglect, and experiences of inconsistency. Of these, only emotional abuse, neglect, and experiences of inconsistency were represented in our eight selected items. An example item is: ‘I felt that someone in my family hated me’. Participants rated items on a five point Likert scale from 1 (never) through 5 (very often). A factor analysis (Principal Axis Analysis; PAA) with the eight items signaled a one factor solution (eigen value: 3.91; explained variance: 48.93%). Cronbach's alpha for these items was.88. In addition, participants were classified as having experienced childhood trauma according to recently reported cut-off scores for Germany [51].

#### Stress reactivity

We selected eight items from the Stress Reactivity Scale [52] to measure stress reactivity that had the highest factor loadings on the general factor. The Stress Reactivity Scale measures general stress reactivity as well as stress reactivity in specific domains (social evaluation, social conflicts, failure, work overload). Moreover, anticipatory stress reactivity, that is, feeling nervous before a previously announced stressor, and prolonged stress reactivity, that is, difficulty relaxing after occurrence of a stressful situation, can be measured with this instrument. Of these, stress reactivity concerning social conflicts and prolonged stress reactivity were the only domains that were not reflected by our choice of items. An exemplary item is: ‘When I′m wrongly criticized by others I am normally annoyed for a long time’. Items are rated by marking one out of three statements, leading to a scale ranging from 0 to 2. According to a PAA, a one-factorial structure (eigen value: 3.09; explained variance: 38.65%) underlies the items used in the current study. Internal consistency for these eight items was.83.

#### Resilience

To obtain a global measure of resilience, we used eight items from the German version of the Resilience Scale [53] with the highest factor loadings. This instrument encompasses aspects of personal competence (e.g., self-esteem) and acceptance of self and life (e.g., flexibility). Items are rated on a seven point Likert scale. An example for an item is: ‘I usually manage one way or another’. A PAA with our eight selected items indicated a one factor solution (eigen value: 3.10; explained variance: 38.80%). Internal consistency was.83.

#### Chronic stress

To measure chronic stress, we used eight items from the screening version of the Trier Inventory for the Assessment of Chronic Stress [35] that had the highest factor loadings. The Trier Inventory for the Assessment of Chronic Stress refers to the past three months, measuring different facets of chronic stress, namely chronic worrying, work and social overload, excessive demands at work, and lack of social recognition on a five point Likert scale. An example item is: ‘I do not have enough time to perform my daily tasks’. The eight items used were based on a common factor (eigen value: 4.57; explained variance: 57.09%). Cronbach's alpha was.91.

#### Functional somatic syndromes

Details on how FSS were diagnosed are reported elsewhere [49]. In brief, we administered a previously developed questionnaire, the Questionnaire on Functional Somatic Syndromes [54]. The German version of this scale is freely available as a Web supplement to the original article (http://content.karger.com/ProdukteDB/miscArchiv/000/333/298/000333298_sm_supplemental_material.pdf). The Questionnaire on Functional Somatic Syndromes consists of three different parts which are connected via several algorithms. First, a screening part encompassing various somatic symptoms was presented. These items represent cardinal symptoms of 17 different FSS. Symptoms were rated according to frequency of occurrence (‘never/rarely’, ‘frequently’, ‘almost always/always’). Second, if participants reported cardinal symptoms that were characteristic of one FSS (e.g., abdominal pain), additional questions based on diagnostic criteria, e.g., Rome III [55], were presented. Third, those who fulfilled the diagnostic criteria of a specific FSS were surveyed about health care visits (e.g., ‘Did you ever visit a doctor about your abdominal pain/changes in bowel movement?’). Participants who responded with ‘no’ were counted as non-cases. Participants who responded with ‘yes’ were ultimately directed to a list of items addressing frequent medical exclusionary diagnoses (‘What diagnosis did your doctor give you?’). Participants were labeled as having an FSS if they reported that no abnormalities which might account for their symptoms (e.g., an inflammatory bowel disease) had been detected by their physician.

#### Mental disorders

Details on how the presence of mental disorders was assessed can be found in our previous report [49]. In brief, we used the German version of the Patient Health Questionnaire [56] to screen for the most common mental disorders, including somatoform syndrome, major depressive syndrome, anxiety syndrome, alcohol syndrome, and bulimia nervosa. All questions and algorithms of the PHQ are guided by DSM-IV criteria [57].

### Statistical analyses

We tested our conceptual model (see Figure 1) using a structural equation modeling (SEM) approach. This approach allowed us to evaluate how well our hypothesized relationships between a latent exogeneous variable (childhood trauma), latent mediators (stress reactivity, resilience, and chronic stress), and a manifest dichotomous endogeneous variable (FSS) fit our data. We used item parceling to form our latent variables [58]-[61]. More specifically, we created two parcels for childhood trauma, stress reactivity, resilience, and chronic stress, with each parcel being based on four items using an item-to-construct balance approach [58]–[61]. In case of unidimensional constructs (see the results of the PAA) the parceling approach is recommended as a method to reduce the number of variables and to improve the stability of the parameter estimates [58]–[61]. As in our study FSS was a dichotomous endogenous variable, we used the modified weighted least squares method (WLSMV) for our analysis [62]. To estimate to what extent the empirical covariance matrix of the involved variables could be reproduced by the model, we conducted a χ^2^-Test and referred to several fit indices: Comparative fit index (CFI), Tucker-Lewis index (TLI), and root mean square error of approximation (RMSEA). A CFI ≥.95, a TLI ≥.95 as well as an RMSEA ≤.05 signals a good model fit [63], [64]. To test the indirect effects for statistical significance, we used the conventional Sobel test [65], [66]. Since the Sobel test does, however, rest on the often implausible assumption that both the sampling distribution and indirect effect is normally distributed, we additionally applied the bias-corrected bootstrapping approach as recommended by MacKinnon et al. [67]. The standard errors of the indirect effects and their 95% confidence intervals were estimated based on 1′000 re-samples. In the results section, we report standard errors and *p*-values based on the Sobel Test (see also Table 1 and 2) and confidence intervals stemming from the bootstrapping approach. All analyses were conducted using MPlus V7.

**Table 1:** Cross-sectional model direct and indirect effects (standardized coefficients) of variables on chronic stress and FSS (T_0_).

	Beta (SE)	*p* value
**Measurement Model**		
Childhood trauma		
Parcel I	.904 (.014)	<.001
Parcel II	.926 (.014)	<.001
Stress reactivity		
Parcel I	.873 (.009)	<.001
Parcel II	.823 (.009)	<.001
Resilience		
Parcel I	.865 (.008)	<.001
Parcel II	.894 (.008)	<.001
Chronic stress		
Parcel I	.925 (.006)	<.001
Parcel II	.938 (.006)	<.001
**Structural Model**		
Effect on stress reactivity		
Childhood trauma	.261 (.019)	<.001
Effect on resilience		
Childhood trauma	−.246 (.016)	<.001
Correlation between stress reactivity and resilience	−.489 (0.16)	<.001
Effects on chronic stress		
Direct effects		
Childhood trauma	.116 (.014)	<.001
Stress reactivity	.582 (.016)	<.001
Resilience	−.188 (.016)	<.001
Indirect effects		
Childhood trauma via stress reactivity	.152 (.012)	<.001
Childhood trauma via resilience	.046 (.005)	<.001
Effects on FSS at T_0_		
Direct effect		
Chronic stress	.268 (.032)	<.001
Indirect effects		
Childhood trauma via chronic stress	.031 (.005)	<.001
Childhood trauma via reactivity and chronic stress	.041 (.006)	<.001
Childhood trauma via resilience and chronic stress	.012 (.002)	<.001
Stress reactivity via chronic stress	.156 (.019)	<.001
Resilience via chronic stress	−.050 (.007)	<.001

FSS  =  functional somatic syndromes.

Beta  =  standardized coefficient.

SE  =  standard error.

**Table 2:** Longitudinal model direct and indirect effects (standardized coefficients) of variables on chronic stress and FSS (T_1_).

	Beta (SE)	*p* value
**Measurement Model**		
Childhood trauma		
Parcel I	.915 (.039)	<.001
Parcel II	.969 (.041)	<.001
Stress reactivity		
Parcel I	.869 (.026)	<.001
Parcel II	.853 (.026)	<.001
Resilience		
Parcel I	.794 (.028)	<.001
Parcel II	.910 (.031)	<.001
Chronic stress		
Parcel I	.913 (.023)	<.001
Parcel II	.897 (.023)	<.001
**Structural Model**		
Effect on stress reactivity		
Childhood trauma	.196 (.050)	<.001
Effect on resilience		
Childhood trauma	−.173 (.048)	<.001
Correlation between stress reactivity and resilience	−.545 (0.44)	<.001
Effects on chronic stress		
Direct effects		
Childhood trauma	.117 (.047)	<.05
Stress reactivity	.433 (.053)	<.001
Resilience	−.169 (.053)	<.01
Indirect effects		
Childhood trauma via stress reactivity	.085 (.025)	<.01
Childhood trauma via resilience	.029 (.012)	<.05
Effects on FSS at T_1_		
Direct effect		
Chronic stress	.339 (.065)	<.001
Indirect effects		
Childhood trauma via chronic stress	.040 (.018)	<.05
Childhood trauma via reactivity and chronic stress	.029 (.011)	<.01
Childhood trauma via resilience and chronic stress	.010 (.005)	<.05
Stress reactivity via chronic stress	.147 (.036)	<.001
Resilience via chronic stress	−.057 (.021)	<.01

FSS  =  functional somatic syndromes.

Beta  =  standardized coefficient.

SE  =  standard error.

## Results

### Sample characteristics

A total number of N = 6′206 participants visited the website and about 51% of them finished the survey. After the exclusion of implausible (e.g., survey response duration below 15 minutes) and incomplete datasets, N = 3′054 datasets remained for further analyses. Of these, 2′042 (73.4%) were women and 812 (26.6%) were men and mean age 24.6±5.6 (SD) years. The majority (92.6%) of the participants was not married, and parental household income was almost uniformly distributed across nine predefined categories ranging from less than 3′000 to more than 10′000 Swiss Francs per month (equal intervals across categories). In our sample, physical neglect (24.7%) and emotional abuse (19.4%) were the most prevalent forms of trauma. The prevalence rates of each FSS are reported elsewhere [49]. The most frequently reported FSS were premenstrual syndrome (112 cases or 5%), functional dyspepsia (57 cases or 1.9%), premenstrual dysphoric disorder (34 cases or 1.5%), hyperventilation syndrome (40 cases or 1.3%), and irritable bowel syndrome (39 cases or 1.3%). In our sample, 15.1% had an alcohol syndrome, 9.0% an anxiety syndrome, 8.1% a major depressive syndrome, 6.5% had a somatoform syndrome, and 1.4% had a preliminary diagnosis of bulimia nervosa. Four hundred twenty-nine participants took part in the follow-up survey at T_1_, and these participants did not differ from those who chose not to participate with regard to gender, marital status, household income, childhood trauma, stress reactivity, resilience, and chronic stress (data not shown). However, this sub-sample was slightly older (25.6±7.0 vs. 24.4±5.3 years; *t*(512.25) = −3.36, *p* = .001). Of the participants at follow-up, 21 out of 429 (4.9%) had at least one newly developed FSS (incident cases) and 10 out of 48 (20.8%) were stable cases reporting at least one FSS.

### Model fit

**Figure 2:**
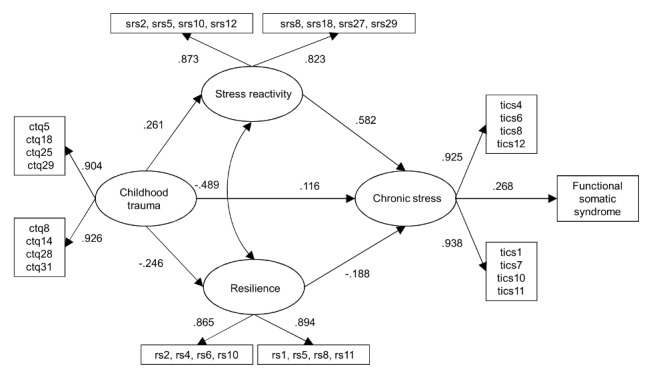
Cross-sectional path analysis model: FSS on chronic stress including standardized path coefficients. ctqx  =  Childhood Trauma Questionnaire, indicator x. srsx  =  Stress Reactivity Scale, indicator x. rsx  =  Resilience Scale, indicator x. ticsx  =  Trier Inventory for the Assessment of Chronic Stress, indicator x.

The first cross-sectional model analyzed included all variables as hypothesized in Figure 1. The model demonstrated good fit statistics (χ^2^(20)  = 50.546, *p*<.001, CFI  = .995, TLI  = .991, RMSEA  = .022, 90% CI [.015.030]). The χ^2^-test was significant for this and the following models, but needs to be interpreted in the context of the large sample size. No significant direct effect of childhood trauma on FSS emerged (Beta  = .083, SE(Beta)  = .050, *p* = .101). We thus removed this path and repeated our analysis. Our second and final cross-sectional model as depicted in Figure 2 fit our data well (χ^2^(21)  = 48.808, *p*<.001, CFI  = .995, TLI  = .992, RMSEA  = .021, 90% CI [.013.029]) and did not have a significantly worse fit than the more complex initial model (Δχ^2^  = 1.74, Δ*df*  = 1, *p* = .19).

**Figure 3:**
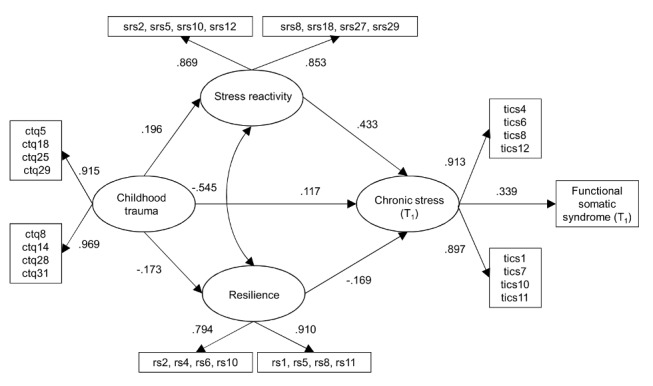
Longitudinal path analysis model: FSS on chronic stress including standardized path coefficients. ctqx  =  Childhood Trauma Questionnaire (T_0_), indicator x. srsx  =  Stress Reactivity Scale (T_0_), indicator x. rsx  =  Resilience Scale (T_0_), indicator x. ticsx  =  Trier Inventory for the Assessment of Chronic Stress (T_1_), indicator x.

In order to evaluate whether our variables were in fact predisposing/precipitating and/or perpetuating factors for FSS, we analyzed our sub-sample of 429 participants that had participated in the follow-up survey. Again, the direct effect of childhood trauma on FSS was non-significant (Beta  = .052, SE(Beta)  = .064, *p* = .410) and therefore restricted to zero (see Figure 3). As it was the case for the cross-sectional version, this more parsimonious model fitted the data well (χ^2^(21)  = 32.676, p<.05, CFI  = .982, TLI  = .969, RMSEA  = .036, 90% CI [.001.059]).

### Model parameters

All parameter estimates for our cross-sectional and longitudinal analyses are shown in Tables 1 and 2, respectively. In accordance with our proposed conceptual model, childhood trauma had a positive effect on stress reactivity and a negative effect on resilience. Unsurprisingly, the parts of stress reactivity and resilience that could not be accounted for by childhood trauma were negatively related (correlation).

As shown in Tables 1 and 2, our results regarding the direct effects of childhood trauma, stress reactivity, and resilience on chronic stress were also in accordance with our assumptions. Exposure to childhood trauma resulted in more chronic stress at both time points. The same was true regarding stress reactivity, whereas being resilient had an opposite effect. Having suffered from chronic stress during the past three months, in turn, significantly enhanced the probability of having an FSS.

In line with our mediation hypotheses, stress reactivity (Beta  = .156, *p* <.001, 95% CI: 0.115, 0.197) had a positive indirect effect on FSS via elevated levels of chronic stress, whereas resilience indirectly lowered the probability of FSS (Beta  = −.050, *p*<.001, 95% CI: −0.067. −0.034) via reduced amount of chronic stress (indirect effects; see Table 1). As can be seen in Table 2, this was also true when chronic stress and FSS were measured at T_1_ (indirect effect of stress reactivity: Beta  = .147, *p* <.001, 95% CI: 0.065, 0.229; indirect effect of resilience: Beta  = −.057, *p*<.01, 95% CI: −0.113. −0.001). Moreover, the hypothesized indirect effects of childhood trauma on FSS via chronic stress (Beta  = .031, *p* <.001, 95% CI:.019,.043), reactivity and chronic stress (Beta  = .041, *p*<.001, 95% CI:.028,.054), as well as via resilience and chronic stress (Beta  = .012, *p*<.001, 95% CI:.008,.017) were significant and in the expected direction. Thus, childhood trauma increased the probability of having an FSS via three indirect routes. The indirect effects of childhood trauma on FSS via chronic stress (Beta  = .040, *p*<.05, 95% CI:.001,.079) and via reactivity and chronic stress (Beta  = .029, *p*<.01, 95% CI:.004,.049) could be replicated in the prospective model (Sobel test and bootstrapping approach). However, the indirect connection via resilience and chronic stress (Beta  = .10, *p*<.05, 95% CI: −.003,.023) was only significant when applying the Sobel test.

## Discussion

We set out to examine the role of stress in FSS both cross-sectionally and prospectively. In accordance with our conceptual model, our data show the occurrence of childhood trauma to be significantly related to elevated stress reactivity and attenuated resilience, which in turn predicted the manifestation of FSS via chronic stress. While we observed an indirect effect of childhood trauma on the development and perpetuation of FSS via chronic stress, we were not able to show a direct link between traumatic experiences and FSS.

Emotional neglect and abuse were the particluar types of trauma that were indirectly associated with FSS in our sample. These types of childhood trauma resulted from an environment that was perceived as unstable, with caregivers not only failing to meet the child's emotional needs but also showing demeaning and humiliating behavior towards the child [20]. We observed that such experiences were associated with higher stress reactivity and lower resilience in adulthood. More specifically, affected individuals reported elevated habitual levels of stress before important tasks, in response to high workload, social evaluation, and experiences of failure [52]. In addition, they had weaker beliefs in their personal competence and lower levels of acceptance of themselves and their lives [29]. Of note, although stress reactivity and resilience were highly correlated in our sample, they cannot be considered as two sides of the same coin. Rather, they seem to exhibit both overlapping and unique aspects in the stress response context, a finding that is mirrored by recent research suggesting specific neurocircuits to underlie resilience [68]. Interestingly, a recent study showed specific regional patterns of cortical thinning depending on whether participants reported childhood experiences of sexual or emotional abuse [69]. Unlike sexual abuse, cortical thinning was present in regions commonly associated with self-awarness and -evaluation in participants reporting emotional abuse [69]. It is thus conceivable that early adverse stimulation of these brain areas contributes to a vulnerable concept of the self, ultimately resulting in altered stress responses when meeting important tasks or when being subject to social evaluation [69].

These findings fit well with the observation that social-evaluative stressors (like public speaking tasks) are highly effective in eliciting a stress response in FSS patients in the laboratory [25], [26]. According to the Lazarus and Folkman framework [19], the reason for higher psychological stress responses lies in the appraisal of one's resources as insufficient when encountering a stressor (‘secondary appraisal’). Moreover, emotional abuse and neglect and a personality profile that is characterized by high stress reactivity and low resilience seem to render individuals vulnerable to the appraisal of future ambiguous stimuli as a threat [19]. In our sample, these persons were in fact characterized by a high amount of chronic stress at a later timepoint, including worrying and feeling overwhelmed with various kinds of demands. In line with this finding, a study among college students found elevated levels of worry and co-morbidity with generalized anxiety disorder to be characteristic of individuals suffering from irritable bowel syndrome [70]. In our sample, chronic stress was accompanied by the development and perpetuation of FSS. Contrary to our expectations, we did not find a direct effect of experiences of childhood trauma on FSS when all other variables were controlled for. Based on our results, one could thus speculate that for the sensorimotor and emotional concomitants of trauma exposure [46] to translate into FSS, the occurrence of chronic stress as a trigger later on in life is a necessary prerequisite. Regarding the perpetuation of FSS, a recent electronic diary study found stress to predict an increase in functional symptoms in 30% of their participants, thus mirroring our findings in a setting with high ecological validity [71].

Our findings regarding a linkage between childhood trauma and altered stress reactivity in FSS can be discussed in the context of biological findings [72]. For instance, Videlock et al. found traumatic childhood experiences to be related to altered neuroendocrine stress reactivity to a visceral stressor in a sample of irritable bowel syndrome patients [73]. This raises the possibility that our results mirror disturbances at a neuroendocrine level. Importantly, this study utilized an acute stressor in the laboratory as a means of eliciting stress reactivity. However, whether chronic stress outside of the laboratory may serve as an ‘opportunity’ to translate dysfunctional stress reactivity into FSS has received little attention so far. A recent study conducted in women with fibromyalgia revealed a shorter gestational length (another indicator of early life stress) to be related to altered neuroendocrine stress reactivity, while at the same time 70% of the sample reported severe stress as a triggering event for their symptoms [74]. Unfortunately, the authors did not report to what extent these events were only present in the early life stress/altered stress reactivity group.

This is the first study examining the association between childhood trauma and resilience in FSS patients. It is only in recent years that the neurobiological basis of resilience in humans has begun to be explored [75] and a mere handful of studies have made an effort to approach the subject of resilience integratively, that is, considering both biological and psychological aspects. For instance, a study in patients suffering from posttraumatic stress disorder demonstrated blunted increases in neuropeptide Y (a stress modulating neuropeptide) in response to a pharmacological stimulant of the stress hormone norepinephrine [76]. These findings are intriguing in light of the fact that neuropeptide Y is discussed as a protective factor in stress regulation [75] and high comorbidities with PTSD are present in many patients suffering from FSS [77], [78]. However, to what extent this finding applies to patients with FSS remains purely speculative at this point, and both psychological and biological aspects of resilience clearly need to be further scrutinized.

Several limitations need to be taken into account when interpreting our study results. First, the present survey was conducted in a student sample that cannot be considered representative of the general population. Second, our approach of establishing diagnoses of FSS was dependent on the reporting of health care visits, which could potentially lead to an underestimation of prevalence rates. Also, due to the nature of a web-based data collection approach, we were not able to confirm these diagnoses through a physical examination or laboratory assessment in our participants. Third, we relied on retrospective self-reported data to measure childhood trauma. Although the CTQ is a well-validated questionnaire that has been used extensively in research on childhood trauma, we are not able to provide external corroboration of our findings (e.g., by simultaneously asking a family member about childhood trauma occurrence).

In conclusion, we provide a comprehensive view on the role of stress and resilience in the development and perpetuation of various FSS. Large-scale epidemiological studies are warranted to replicate our prospective findings in the general population. Also, while our data suggest stress to be a risk factor that is common to several different FSS, further work is required to confirm our model in samples of specific FSS. Finally, there is an urgent need for integrative research acknowledging both biological and psychological aspects of stress reactivity and resilience in the search for the pathophysiology of FSS.

Our findings have important clinical implications. First, we advocate that attention be paid to the possibility of childhood trauma in FSS patients and affected individuals be offered adequate treatment. Second, given our results, patients with FSS are likely to benefit from interventions reducing stress reactivity (e.g., by learning relaxation techniques) and/or enhancing resilience (e.g., by strengthening individual resources). Finally, due to the observation that the incidence and maintenance of FSS is dependent on chronic stress, a case can be made for psychological therapy as a means of improving stress management strategies.
